# The Sickness and Mortality of the Federal Volunteers

**Published:** 1863-01

**Authors:** 


### Art. IX.—THE SICKNESS AND MORTALITY OF THE FEDERAL VOLUNTEERS.

In our lats impression we discussed at some length a remarkably interesting and instructive series of documents, published by the United States Sanitary Commission, on the health-condition of the Federal Volunteers. From these papers we learned the sanitary history of this gigantic force from the time of its first formation until near the termination of 1861. Papers subsequently received now enable us to extend this history to March, 1862. On the 18th of May last, Mr. Elliott, the actuary to the Sanitary Commission, laid before the Commission a preliminary report on the mortality, sickness, and other casualties of the volunteer forces from the commencement of the war to the period mentioned. This report is far from being perfect, "many regiments" having neglected to make returns. Moreover, the experience of three months' volunteers is not included. The reason of this exclusion is not precisely stated, but it is to be presumed that volunteers of this standing enter into the category of "home guards, and other bodies of men not in active service," who are not noted in the returns. Again, deaths which have occurred among those discharged from or otherwise quitting the service are not taken into account, there being as yet but little or no information concerning the mortality of this class on record. For the rest, taken with due reservation, the returns are of great interest.

It would appear that, from the beginning of the war to the 1st of March, 1862, the annual rate of mortality, among those portions of the volunteer forces which had made regular returns, was fifty-three (53·2) per 1000 of the numerical strength. Of this proportion of deaths, about forty-four (44·6) were occasioned by disease and accident, and about nine (8·6) from wounds received in action. The death-rate among the

---

commissioned officers was thirty-three (33·2) per 1000; among the men fifty-four (54·0).

These are the results of the actual returns, but, as we have already intimated, they are doubtless understated. If, however, the rate of mortality experienced by those who quit the service (by discharge for disability, desertion, or otherwise,) is the same as those continuing in the service, to wit, 53 per 1000—a very moderate estimate, Mr. Elliott thinks, since those discharged for disability are justly presumed to be at the time, in point of health, inferior to their comrades who remain—the estimated annual rate would, according to that gentleman, differ little from 65 per 1000.

A further analysis of the returns shows that the mortality of *officers* from wounds received in action was about *one-third* (more exactly, 34 per cent.) *greater* than those of the *men;* while the mortality from disease among the former was only about *one-half* (48 per cent.) of the latter; but the liability to death among the officers from all causes (both disease and violence) is nearly *two-thirds* (61 per cent.) that of the men.

Again, *two-thirds* of the deaths of officers and *five-sixths* of those of the men resulted from disease and accident; the remaining one-third and one-sixth respectively being caused by wounds received in action. During the Crimean war seven-eighths of the mortality of the British troops arose from disease, and one-eighth from violence, which term includes deaths from accident and from wounds received in action.

A steady increase in the general mortality of the forces from the commencement of the war is exhibited by the returns. The death-rate for the autumn months was about twice as great as that for the summer period, and the winter rate, in turn, doubled that of the autumn. Some portion of this increased rate is apparent only, and is to be attributed to the greater perfection of the later returns.

The volunteers are divided, in Mr. Elliott's report, into three categories, the first including the troops recruited in the New England States; the second including those from the Middle States (to wit, the Middle States proper—the States of Delaware, Maryland, and Virginia); and the third including those from the Western States (with which are joined the States of the South-west). The mortality of the forces recruited in the Western States, and which, as a rule, operated in the West, was almost precisely three times greater than that of the troops recruited in the Middle and New England States, and which as a rule serve with the army in the East. The death-rate from wounds received in action in the Western army was five times, and that from disease nearly three times, as great as the corresponding rates among the forces serving in the East.

A like contrast was also observed in the *sickness-rate* of the troops East and West. The rate of the latter (161 per 1000) was more than twice that of the former; the average constant sickness-rate for the *entire* army being 104 per 1000 strength.

The constant sickness-rate among the commissioned officers was only two-thirds that among the men. The rates of constant sickness, like the rates of mortality, increased in the autumn and winter.

The excess of the rates of sickness and mortality in the Western forces over those of the East is attributed—"in no small degree, not merely to the greater activity of the former in the field, to over-exertion and exposure, as the result of severe and long-continued marches, and to stubborn and deadly encounters with the enemy in arms, but also to badly chosen camp sites, to imperfect and neglected drainage, (the nature of the surface and soil not unfrequently being such, that suitable camp sites free from malaria and affording ample facilities for drainage could not be found, if sought), to the too crowded condition of hospitals, to less variety in food (soft bread and desiccated vegetables, in very many Western regiments, being seldom or never had), and to less of skill and care in its preparation; to water of impure quality, and sometimes of insufficient quantity; to the greater disposition, on the part of the soldiers, to neglect appliances for personal comfort; and to the greater neglect of, or lack of means for, enforcing cleanliness."

The average strength of the regiments throughout the entire force was 872, officers and men, of which 91 were constantly sick. The rate of recruiting, in excess over discharged for expiration of service, during the nine months from June 1st, 1861 to March 1st, 1862, was 230 per 1000 enlisted men.

To supply the continuous *losses* in the ranks, other than losses arising from expiration of service, required recruits at the rate of nineteen (19·1) per 1000 per month; or 229 per 1000 per annum. Of the last-mentioned proportion, 54 was the number required to supply the annual loss from deaths; 100 the annual loss from discharges; 14 the loss from missing in action, not afterwards accounted for; 50 the annual loss from desertion, the deserters not subsequently returning to duty; and 11 the loss from other causes, specified and unspecified. To supply a constant force of 500,000 *effective*, that is to say, healthy and able men, in the field, at the rates of loss thus experienced, it would be requisite to maintain, "in hospitals or elsewhere," an additional force of 58,300 men, making the entire force maintained 558,300

men, 22,000 (4 per cent.) of whom would be commissioned officers, and 536,000 (96 per cent.) privates. Further, since to supply continuous losses in the ranks, other than losses from expiration of service, requires recruits at the annual rate of 229 per 1000, it follows, that to keep the ranks of the 536,000 constantly full, would require annually 123,000 recruits, 29,000 being demanded to supply the annual loss from deaths; 54,000 the annual loss from discharges on account of disability; 27,000 for excess of desertions over returns of deserters to duty; 7000 for missing in action, not subsequently otherwise accounted for; and 6000 the loss from other causes.

The 58,000 sick requisite to be maintained to secure, at the before mentioned rates of sickness, an effective force of 500,000 men, are equivalent to 67 regiments of average numerical strength—the entire force of 558,000 men to be maintained being equivalent to 640 regiments.

These calculations exhibit in a very clear light the enormous and most costly influence which sickness exercises over the efficiency of an army in the field. And in this case the illustration is the more striking, since the sickness-rate (104 per 1000) of the Federal volunteers within the period under examination contrasts very favourably with the campaigning experiences of other armies. The average constant sickness-rate of the British army in Spain and Portugal from 1808 to 1814 was 209 per 1000; and that of the French army in the Peninsular war was, in Spain 136, and in Portugal 146, per 1000.

One or two additional items of comparison, cited by Mr. Elliott, may be briefly noted.

The average annual rate of mortality for the army of the United States in times of peace is 26 per 1000. The mortality of the troops engaged in the Mexican war (1846-'47-'48) was at the annual rate of 118 per 1000—104 from disease and accident, 14 from wounds in action. This rate is about two and one-fifth times greater than that experienced by the present volunteer forces prior to May, 1862. The Mexican forces consisted of regulars and volunteers, the former embracing the "old establishment" of 15,734 men, and an "additional force" of 11,186 men. The volunteers numbered 73,532. The death-rate in the "old establishment" amounted to 104 per 1000 per annum; in the "additional force" to 162; and in the volunteers to 116. In the "old establishment" 23 per cent. of the deaths arose from wounds received in action, as compared with 10 per cent. among the volunteers and "additional force." The large preponderance of the mortality from disease, over that occurring in the "old establishment," among the raw soldiers of the additional force and the less perfectly trained volunteers, and the comparatively small amount of mortality from wounds received in action among the two latter forces, is very significant. Equally significant and instructive are the facts that the proportion of men *discharged* from service for disability and by civil authority, was, among the volunteers, 150 per 1000 per annum; among the "additional force," 63·1; and among the "old establishment," 63.

During the war in the Peninsula, in 1811-14, the annual death-rate among the British troops was 165 per 1000 strength —113 being from disease and accident, and 52 from wounds received in action. The annual rate of mortality experienced by our troops during the Crimean war was 232 per 1000. In the first three months of 1855, the death-rate was no less than 912 per 1000 per annum, and in the January of that year it reached 1174. In the three months terminating the war, the annual death-rate was but 16 per 1000.

Although no connected history, or series of official documents, throwing light upon the history of the sanitary events of the late Federal campaign, since the period of Mr. Elliott's report, is as yet forthcoming, or, at least, is accessible in this country, we are not left altogether in the dark on the subject. The number of the *Revue des Deux Mondes* for the 15th October last contains a singularly interesting account of the campaign of the Army of the Potomac between March and July, 1862. This account is signed "M. A. Trognon," but it embodies the experience, and is doubtless from the pen, or else is drawn up from detailed notes, of one of the two Orleanist princes who served throughout this campaign on General McClellan's staff. The account deals chiefly with the military aspects of the campaign, but in many parts it illustrates the correctness of the statements of the Sanitary Commission, on the defective training, inaptitude, and ignorance of the officers of the volunteer forces, and the evils arising from these sources. The author states that the Federal Government had succeeded in collecting, as it were by enchantment, vast masses of men and an immense materiel; but it had not been able to create by vote, discipline, obedience, and hierarchical respect, without which the masses were armed mobs, not armies. "L'obéissance dans cette armée," he writes, "ressemble à l'obéissance que des enfans qui jouent au soldat montrent à celui de leurs camarades qu'ils ont fait leur capitaine." As the Sanitary Commission predicted, this evil, than which none could be more serious in active warfare, exaggerated all the other evils of campaigning, and formed an incubus upon the movements and combined operations of the forces, which rendered in a great measure nugatory individual bravery, however conspicuous, and interposed an almost insuperable obstacle to ultimate success. The army of the Potomac advanced upon Richmond through a flat country, covered with marshy woods, traversed by few roads, and these hardly worthy of the name, and at a time when intense heat alternated with heavy rains. It was merely necessary for the Confederates to hold the Federals in check within this district, and the discomfiture of the force would be quickly insured by the natural influence of the locality upon the health of the men, unless lavish reinforcements and supplies were forthcoming. Both the former and the latter were doled out to General McClellan with the most niggard hand; and his reinforcements were entirely insufficient to fill up the gaps occasioned by sickness and death.

The Federal forces were disembarked at Fort Monroe in the beginning of April, and late in May they reached the vicinity of Richmond. In a note appended to Mr. Elliott's report, we learn how heavily the check to the advance experienced there affected the sanitary welfare of the troops. He states that the average number of sick men present with the Army of the Potomac, on the 10th June, 1862, was 57 per 1000 (including the present and the absent); and that 214 per 1000 of the entire force were absent—208 being absent with authority, and 6 without authority. "Assuming one-third of the absent to be sick," he adds, "a rate not inconsistent with previous experience, it follows that 128 per 1000 of the Army of the Potomac (present or absent) was then sick, a rate about one-fourth greater than the average rate indicated by the returns for the entire army for the nine months ending the 1st of March previous—and about seven-tenths greater than such experience of the regiments from the New England and Middle States."

For the rest, the story of the disastrous results of this campaign of the Federal forces before Richmond, as well as of the Federal armies generally, has been told so well and instructively by a Transatlantic contemporary,* that we shall quote the major portion of his account, as the original may not be accessible to many of our readers. He writes:—

"The secret of the failure of our armies is comprised in this single word—SICKNESS. Study the last campaign in whatever light we may, the inevitable conclusion is that our defeats are almost solely due to sickness. Of the entire army, one hundred regiments are to-day invalided, and this represents but a fraction of the actual reduction of its physical energy and strength. The campaign on the Peninsula is a striking example of the utter failure of a large and well-appointed army to accomplish

* *American Medical Times,* Aug. 23, 1862.

its purpose, when little or no regard is paid to the prevention of disease. Within the short space of three months it is estimated that 50,000 men were sent to the rear of the 'Grand Army of the Potomac.' During that time it was within twelve hours' sail of the Capital, and commissary stores were in unlimited quantities at the command of the proper officers. The army marched less than a hundred miles through a rich farming country, but long before it reached the point for effective operations the commanding officer was obliged to ask to have his army renewed. During this time there was no prevailing epidemic, or other disease which a careful attention to the simplest laws of hygiene would not in a great degree have prevented.

"Let us notice some of the more palpable causes of weakness and disease. The first cause of weakness is due to mustering into service of unfit persons. This has been done to a most dangerous extent. Boys and old men, men suffering from hernia, varicose veins, chest and heart diseases, &c., often passed muster without a word of objection. There are many instances of persons joining the army because their diseases incapacitated them for active business. The blame here rests with the medical inspectors appointed by the State authorities.

"These inspectors were in some instances utterly unfit for such duty, being unqualified to make a proper medical inspection. At one of the most important recruiting stations in the country nearly every form of disability was overlooked. In other instances, the inspectors, for the smallest bribes, passed men whom they knew to be unfit for the service. An army made up of such material has, within its very earliest organization, the seeds of disaster and ultimate failure. The army of the Potomac was composed of much of this material, which was not very apparent while in camp, and subjected to no other fatigue than the daily drill. But the first decided movement diminished the number of soldiers, but not the strength of the army, by thousands. This class of persons have also filled the hospitals throughout the entire season. The first exposure to the inclemencies of the weather and to fatigue has invalided them for the period of their enlistment.

"A second palpable cause of disease has been the unhealthy location and inadequate provision of camps. There is doubtless an occasional military necessity for the location of a camp on grounds unfit for the residence of man, but this is seldom, and almost never of long duration. And yet the army of the Potomac was scarcely ever on healthy grounds, though often in the immediate vicinity of healthy localities. When it broke camp in March the troops were marched rapidly to Manassas, thence back to the Potomac, upon the banks of which they lay for three weeks waiting for preparations for the expedition to be completed. During that time they were almost within sight of their comfortable but deserted tents, with no other covering than meagre shelter tents. As a consequence, the hospitals of Alexandria and every available building were filled with soldiers suffering from rheumatism, pneumonia, pleurisy, &c. When at length the army moved to the Peninsula, the most unhealthy localities were selected. The extensive low grounds at the mouth of James River, that during the spring season are covered with pools of stagnant water, were occupied by the army. Here they remained for weeks with the same meagre tents, and the same result followed as at Alexandria. The hospitals were soon crowded with every form of disease dependent upon exposure; so excessive was the sickness, that hospital tents in larger numbers were erected in the vicinity, and all were crowded. Here the Grand Army had its strength sadly reduced. Moving forward to the scene of its first operations, the army sat down before Yorktown. Here the camping grounds were selected with no better care, and combined with this fertile source of disease was hard labour in the mud and cold. From this point the transports were busily engaged for weeks in conveying the sick away to distant hospitals. The depletion of the force here was so great as to excite apprehension that this army might not be able to cope with its adversary. After the evacuation of Yorktown, the army pressed on by hurried marches to the Chickahominy, where it again sat down in the very stench of malaria. The reduction of its numerical and physical strength now became frightful and alarming. It was rapidly melting away in the very face of the enemy beyond all power of recuperation. In the final struggle its commanding general required 50,000 more men than he had, but that precise number had been invalided from its ranks through gross neglect of their health and comfort.

"A third cause of disease has been a disregard of camp police. Cleanliness of the ground and cleanliness of the person have generally been most shamefully neglected, and the result has been diseases of the severest type. In vast numbers of regiments of the Grand Army, every form of nuisance has been allowed to accumulate on the ground and in and around the tents. In these regiments personal neatness was not even thought of. No trite apology of 'military necessity' can excuse a neglect of cleanliness—the first element of health.

"A fourth cause of disease has been improper and badly cooked food. The supply of food in the gross to the army has not, we believe, been deficient, except in extraordinary cases.

Government has been lavish in this respect, and has not spared money to give a good supply of food. But she has allowed the most consummate knaves to make the purchases, and so fearfully have they imposed upon her confidence, that the term commissary is a by-word and a hissing in the army. Every article that could be adulterated has been—so thoroughly, that of many articles the original cannot be detected by the senses. And yet for all these stores the Government has originally paid the highest market price. In regard to the cooking, it is safe to say that the food could not well be made more unpalatable and indigestible. An hour spent at surgeon's call will convince any one of the truth of this statement; the greater number of those suffering slight indisposition complain that they 'cannot keep the food on their stomachs.'

"These are some of the more prominent features of the medical history of the Grand Army, which our military authorities should study well before they enter on another campaign."

This requires no comment.

The following facts are culled from papers of the Sanitary Commission, dated the 24th September and 21st October, 1862. The number of wounded in the successive weeks of battle before Richmond, at Bull Run, and in Maryland, is stated to have been from eighteen to twenty-one thousand. After the action in Maryland, the supplies of the Sanitary Commission, consisting of twenty-five wagon-loads of stimulants, condensed food, medicines, and conveniences, reached the battle-field *two days in advance* of any other supplies, beyond the small amount in the nearly empty storehouse of the army medical purveyor. The number of wounded then to be provided for amounted to from seven to ten thousand. So little had the disastrous lesson on the Potomac been heeded, that during a movement of General McClellan in Maryland, in presence of the enemy, everything was carried away to the new camp ground within two hours, *except the medical stores*, which remained a solitary pile in the midst of a deserted camp for nearly twenty-four hours, awaiting transportation. Nay more, the Sanitary Commission state, *apropos* of the battle of Antietam, in an appeal for additional help from the public, dated 21st October, 1862, that "there were thirty regiments of one State alone which went into this battle absolutely without the smallest particle of medical or surgical stores in the hands of their surgeons;" and "that the Government supplies sent for their relief did not reach the ground till the third day after the battle." They add: "The recent battles East and West have completely exhausted the reserved stock of the Commission, and it is found now not only impracticable to accumulate supplies, but impossible to meet even urgent demands daily made by hospitals, within sight of the very dome of the Capitol, and growing out of needs the existence of which, as has been well ascertained, casts no censure on the faithfulness and efficiency of the surgeons in charge."

---

### ART. X.—ON THE UNCONSCIOUS LIFE OF THE SOUL.*

